# An Unusual Cause of Splenic Rupture: Burkholderia pseudomallei Infection

**DOI:** 10.7759/cureus.80901

**Published:** 2025-03-20

**Authors:** Muneeba Moin, Mohamed Shoaib, Zeyad Faoor Medhat Alrais, Hawra Ali

**Affiliations:** 1 Critical Care, Rashid Hospital, Dubai Health Authority, Dubai, ARE; 2 Clinical Pharmacy, Rashid Hospital, Dubai Health Authority, Dubai, ARE

**Keywords:** burkholderia, crtitical care, infectious, melliodosis, sepsis, septic shock, splenic abscess, whitmore disease

## Abstract

Melioidosis is an infection caused by the microorganism *Burkholderia pseudomallei*. Melioidosis can affect almost any organ system in the body and can mimic multiple diseases. One of the rare complications caused by this organism is splenic rupture. If left untreated, this infection and its complications can be fatal. We report a case of melioidosis and related complications in our medical intensive care unit which was successfully identified and treated at an early stage resulting in patient survival. This case emphasizes the importance of vigilance in suspecting, diagnosing, and treating melioidosis and its complications in a timely manner.

## Introduction

Melioidosis, also known as Whitmore disease, is an infection caused by gram-negative bacteria, *Burkholderia pseudomallei*. This infection is commonly seen in tropical climates with heavy rains like Southeast Asia and can also be seen in the Middle East, China, and India [1]. The mode of transmission in humans is through direct contact with contaminated sources such as soil or water. It has been noted that the incidence of the infection has increased with increased travel [2]. 

Melioidosis is commonly seen in the fourth or fifth decade of life and is common in patients with diabetes, renal failure, and other forms of immunosuppression [2]. One of the strongest risk factors for the infection is diabetes mellitus with one study showing an incidence of as high as 50% [3]. The infection can manifest in multiple ways in humans such as pneumonia, skin infections, and septic shock. It has a mortality rate of almost 90% if disseminated septicemia is present [4]. Some of the common complications of melioidosis include septicemia, disseminated abscesses (including splenic), meningitis, osteomyelitis, and pneumonia resulting in acute respiratory distress syndrome (ARDS). Splenic abscess is one of the rarest complications of melioidosis with one study stating the incidence as being between 0.2-0.7% [5].

## Case presentation

A 41-year-old male patient, a known case of uncontrolled diabetes mellitus II, not on medication and hyperthyroidism on carbimazole, presented to the emergency department with complaints of a two-month history of on and off fever, associated with night sweats without any associated weight loss. The patient’s last history of travel was about two months prior to presentation and he did not have any recent history of sick contact. He denied alcohol consumption and smoking.

**Figure 1:**
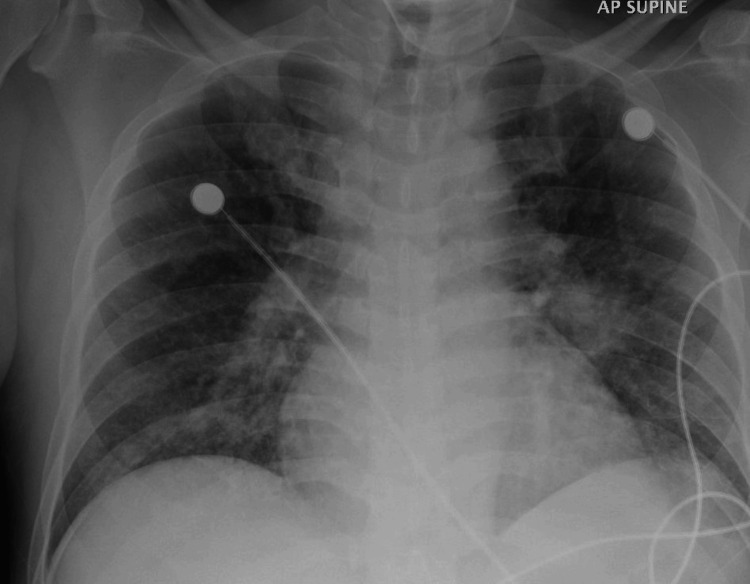
Chest X-ray at presentation

On examination in the emergency department, the patient was noted to have a high-grade fever of 39.4˚C and hypotension (89/52 mmHg). He was also desaturating on room air with an oxygen saturation of 90%. Abdominal examination was unremarkable without any organomegaly, and auscultation of the chest revealed coarse crepitations bilaterally. He was in severe respiratory distress and was also noted to be in septic shock (hypotension did not respond to fluids) for which he had to be started on vasoactive medications (norepinephrine). His chest X-ray showed bilateral hilar and lower zone opacities, more on the right side compared to the left (Figure 1).

Laboratory investigations (Table 1) revealed pancytopenia, deranged renal function (high creatinine and urea), elevated inflammatory markers (C-reactive protein and procalcitonin), and glycated hemoglobin (HbA1C) of 12.9. Pancultures including blood culture were sent. Viral serology including HIV antigen and antibody were negative. A nasal swab for severe acute respiratory syndrome coronavirus 2 (SARS CoV2) tested by polymerase chain reaction (PCR) was also negative. Other respiratory screenings including viral screen, *Legionella* antigen, and *Mycoplasma* antibodies were all negative. The tuberculosis (TB) workup, which included PCR and sputum culture for acid-fast bacilli (AFB), was negative (Table 2).

**Table 1:** Laboratory investigation results

Parameters	Reference Ranges	Patient Values				
		DAY 1	DAY 5	DAY 10	DAY 50	DAY 100
WBC count	3.6-11.0 10^3/uL	2.2	18.5	9.5	8.2	7.8
Hemoglobin	11-16gm/dL	12.3	8.5	7.6	9.2	11.4
Platelet count	150-410 10^3/uL	54	58	138	398	547
Creatinine	0.70-1.20 mg/dL	1.58	1.98	5.79	4.48	0.71
Urea	12-40mg/dL	69	85	129	105	22
C-reactive protein	<5.0mg/L	399.1	301	211	31	13
Procalcitonin	<0.05ng/ml	44.19	17.11	3.96	0.51	0.02
Total bilirubin	0-1.0mg/dL	2.2	39.5		1.35	0.52
Alanine transaminase	0-41 U/L	121	111		35	21
Aspartate Transferase	0-40 U/L	285	101		54	49
Akaline Phosphatase	40-129 U/L	136	247		162	129

**Table 2:** Viral and bacterial serology results PCR: polymerase chain reaction; AFB: acid-fast bacillus

Other Lab Tests	Results
Tuberculosis PCR	Negative
Sputum AFB	Negative
HIV antigen and antibody	Negative
*Legionella* antigen	Negative
*Mycoplasma* antibodies	Negative

The patient’s oxygen requirement was noted to be increasing as he was shifted from high concentration oxygen mask to non-invasive ventilation and he was eventually intubated for type 2 respiratory failure as his blood gas showed hypoxia and hypercapnia and he was noted to be in acute respiratory distress syndrome (ARDS). The patient was then shifted to the ICU for further management.

In the ICU, the patient required high-dose vasopressor support, and antibiotic coverage was optimized with meropenem, azithromycin, and anidulafungin empirically as he was in severe septic shock and further blood work was done (Table 1). Blood culture was noted to be positive for gram-negative rods 15 hours after collection. On day 1 of admission to the ICU, the patient was started on continuous renal replacement therapy for metabolic acidosis and hyperkalemia that did not respond to conservative management. Blood cultures were noted to be positive for *B. pseudomallei *on day 3 of admission (culture sent on day 1 of admission) (Table 3). The organism also grew from respiratory culture. Azithromycin and anidulafungin were later stopped (once blood culture report identifying the organism was released). Meropenem was continued as it is the drug of choice for neuromelioidosis, which could not be ruled out in this patient as lumbar puncture was contraindicated due to thrombocytopenia. Ceftazidime/avibactam and trimethoprim/sulfamethoxazole were also added to the antibiotic regimen on day 6 as blood cultures were persistently positive for the organism despite being on meropenem. All antibiotics were adjusted as per renal replacement therapy guidelines: meropenem 2 gm every 12 hours, ceftazidime/avibactam 2 gm every eight hours, and trimethoprim/sulfamethoxazole 2 gm every eight hours.

**Table 3:** Culture test results

Cultures	Results
Blood (Day 1)	Burkholderia pseudomallei
Blood (Day 5)	Burkholderia pseudomallei
Blood (Day 9)	Burkholderia pseudomallei
Blood (Day 11)	Burkholderia pseudomallei
Blood (Day 16)	No Growth
Respiratory (Day 1)	Burkholderia pseudomallei
Fluid (pus from splenic abscess)	Burkholderia pseudomallei

**Figure 2:**
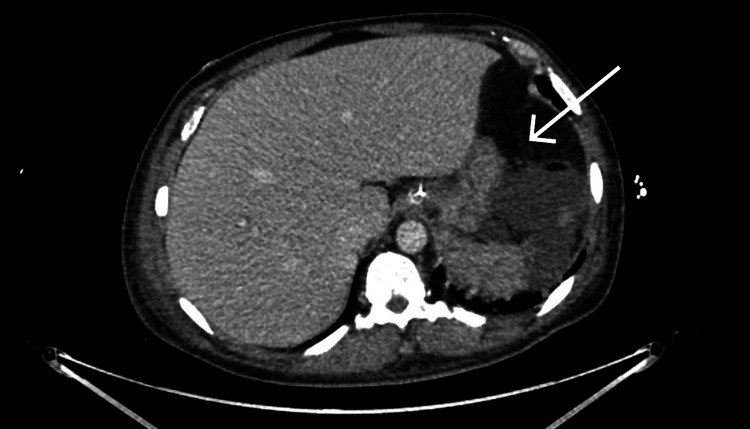
Computed tomography (CT) abdomen with contrast, arrow showing splenic abscess

The patient underwent computed tomography (CT) brain to rule out any complications of melioidosis, and CT chest, abdomen, and pelvis was ordered to investigate the worsening liver function tests and also to rule out possible complications of the infection. The CT brain with contrast was normal but the CT abdomen (Figure 2) revealed a ruptured splenic abscess with subcapsular collections, multiple cavitating pulmonary nodules representing pulmonary septic emboli, and signs of acute kidney injury.

The patient was referred to the surgical service immediately and taken to the operating room for an urgent lifesaving laparotomy which resulted in splenectomy, and abdominal washout was also done. Intraoperatively, the abscess was mainly located at the hilum of the spleen with around 150 ml of pus in situ and around 500 ml of infected ascitic fluid in the abdominal cavity. Dense omental lesions to the spleen were also noted. Samples from the splenic abscess were taken during the surgery and sent for culture which revealed *B. pseudomallei*. The patient's abdomen was kept open, and he went for a relook surgery after three days during which the abdomen was closed.

Serial blood cultures were sent every three to five days and they were persistently positive with clearance being noted on day 16. The patient also underwent a transthoracic and transesophageal echocardiogram which revealed a normal ejection fraction and no evidence of clots of vegetation.

Once the patient improved hemodynamically and his respiratory condition stabilized, he was weaned off mechanical ventilation and eventually extubated on day 12 of admission with a post-extubation Glasgow Coma Scale score of 15/15. During the ICU stay, the patient developed dusky discoloration of all his fingers and toes, which was attributed to the high inotropic support, and was treated conservatively by the vascular surgery team while in the ICU; however, later, this required amputation.

The patient was shifted out of the ICU to the high-dependency ward after 45 days. He completed meropenem for five weeks after which it was stopped (lumbar puncture done on a later date was negative for the organism), trimethoprim/sulfamethoxazole was stopped after five weeks of therapy as the patient suddenly developed an allergic reaction to the medicine (severe rash and itching). He completed two months of ceftazidime/avibactam. He received pneumococcal and meningococcal vaccinations post ICU stay and was overall considered to be improving. An improvement was noticed in renal parameters as well and he did not require any further dialysis. 

Unfortunately, while in the general ward, the patient required amputations of several fingers and toes and was being followed up by the general surgery and wound management teams. He was discharged from the hospital in a stable condition after 105 days of admission. He was discharged on oral doxycycline, to be continued for six months as the eradication phase.

## Discussion

*B. pseudomallei *is an aerobic, non-spore-forming, straight, slender, gram-negative bacillus with cells that range from 1 μm to 5 μm long and 0.5 μm to 1 μm wide motile [6]. This organism, being a facultative intracellular organism, invades and replicates inside polymorphonuclear leukocytes, macrophages, and some epithelial cell lines [7]. People can acquire melioidosis through contact with contaminated soil and surface waters by percutaneous inoculation, aerosol inhalation, or the ingestion of contaminated water or food [8]. Various factors like inoculating dose, mode of infection, host risk factors, and probably differential virulence of infecting *B. pseudomallei *strains can have their influence over the incubation period. The onset of melioidosis within 24 hours has been seen in presumed aspiration after near drowning and in some cases, after severe weather events [9].

An emerging cause of disseminated melioidosis is the increase in air travel. The number of cases has been noted to be increasing among travelers, especially to Southeast Asia and Australia [10]. The main risk factor that was noted in the current patient was uncontrolled diabetes mellitus with an HbA1C of 12.9, which contributed to an increased susceptibility to melioidosis; this finding was also reported by Chen et al. [11] and Basheer et al. [12] in their case reports. Another risk factor as mentioned above, was his recent history of travel to Southeast Asia (the Philippines).

Our patient was treated with multiple antibiotic therapies, including meropenem and later ceftazidime/avibactam and trimethoprim-sulfamethoxazole were added. Ceftazidime was continued for eight weeks which was required in the case of our patient as he was inflicted with severe disease. Studies have shown that the intensive phase can be anywhere between 10 to 14 days but a longer intensive phase (more than four weeks) is preferred for more severe disease resulting in disseminated infection with septic shock and splenic abscesses [13]. As our patient developed acute kidney injury, early renal replacement therapy was initiated, and eventually his renal function normalized and he did not require any further follow-up with nephrology as an outpatient. Lifesaving splenectomy was done in view of splenic abscess and severe septic shock; his abdomen was kept open and closed in the operation theatre after three days. Culture of the splenic abscess was a very important factor in differentiating between melioidosis and tuberculosis since both can have similar presenting features and melioidosis can go into a chronic phase like tuberculosis [14]. He was vaccinated as per the protocol for post-splenectomy patients. Melioidosis can cause septic emboli in multiple organs as was seen in our patient in the form of septic pulmonary emboli and possibly the digital gangrene in both upper and lower limbs could also be attributed to this [15]. After completion of the intensive phase, patients need to be continued on an eradication phase with either trimethoprim/sulphamethoxazole for a minimum of three months which can go up to six months [13].

## Conclusions

B. pseudomallei is a potential causative organism of splenic abscess in patients with risk factors such as diabetes mellitus. Such cases usually do not respond to standard antibiotics and need targeted antibiotic therapy followed by a long eradication phase. A positive blood culture is diagnostic for melioidosis. Appropriate antibiotics and surgical management splenectomy could be lifesaving in severe cases. The aim of this case report is to highlight the importance of timely diagnosis and management along with a high index of suspicion for melioidosis when patients present with classic symptoms as this infection is known as the great mimicker and can be easily missed in favor of other diagnoses, which can prove to be fatal for patients.
